# SYN023, a novel humanized monoclonal antibody cocktail, for post-exposure prophylaxis of rabies

**DOI:** 10.1371/journal.pntd.0006133

**Published:** 2017-12-20

**Authors:** Tzu-Yuan Chao, Shiqi Ren, Enyun Shen, Susan Moore, Shou-feng Zhang, Li Chen, Charles E. Rupprecht, Eric Tsao

**Affiliations:** 1 Synermore Biologics Co., Ltd., Taipei, Taiwan; 2 Beijing Cotimes Biotech Co., Ltd., Beijing, China; 3 Kansas State University Rabies Laboratory, Manhattan, Kansas State, United States of America; 4 Laboratory of Epidemiology and Key Laboratory of Jilin Provincial Zoonosis Control and Prevention, Military Veterinary Research Institute, Academy of Military Medical Sciences, Changchun, China; 5 LYSSA LLC, Atlanta, Georgia, United States of America; Universidad Nacional Mayor de San Marcos, PERU

## Abstract

Rabies is a neglected zoonotic disease that is preventable in humans by appropriate post-exposure prophylaxis (PEP). However, current PEP relies on polyclonal immune globulin products purified from pooled human (HRIG) or equine (ERIG) plasma that are either in chronic shortage or in association with safety concerns. Here, we present the development of an antibody cocktail, SYN023, made of two novel monoclonal antibodies (MAb) CTB011 and CTB012 that could serve as safer and more cost-effective alternatives to the current RIG products. Both CTB011 and CTB012 are humanized MAbs that bind to non-overlapping epitopes on the rabies virus (RABV) glycoprotein (G) with sub-nanomolar affinities. Sequence analysis revealed that many of the critical residues in binding are highly conserved across different species of lyssaviruses. When combined at a 1:1 ratio, CTB011/CTB012 exhibited neutralization capabilities equivalent or superior to HRIG against 10 North American street RABV isolates *in vitro* and 15 prevalent Chinese RABV strains in animal models. Finally, SYN023, at a dosage of 0.03 mg/kg, was able to offer the same degree of protection as standard HRIG administration (20 IU/kg) in Syrian hamsters challenged with a highly virulent bat (*Tadarida brasiliensis*) RABV variant. Taken together, the high-potency and broad-spectrum neutralization demonstrated by SYN023 make it an effective candidate for human rabies PEP consideration.

## Author summary

Rabies is a preventable viral disease and yet human rabies is still prevalent in many less-developed parts of the world. In those regions, the majority of human rabies deaths has been attributed to the low availability and poor accessibility of human antibodies (human immunoglobulin, HRIG), which can only be obtained from healthy individuals who volunteer to receive rabies vaccination. Although a cheaper alternative of equine origin (equine immunoglobulin, ERIG) is more readily available, it is not without safety risks and complications. HRIG and ERIG are effective in treating human rabies because they contain highly diverse antibodies that block different strains of rabies viruses from entering their pathogenic life cycle. To provide a similar spectrum of protection, we identified two antibodies, CTB011 and CTB012, which target conserved regions of the rabies viral particle across a wide range of strains. The 1:1 mixture of the two antibodies, SYN023, was demonstrated to have similar potency to HRIG in animal models. SYN023 therefore shows promise as a safer and more sustainable replacement of HRIG that can be massively produced to save lives.

## Introduction

Rabies virus (RABV) causes an acute, progressive encephalitis with the highest case fatality of any infectious agent [1]. Annually, this zoonosis is estimated to cause in excess of 60,000 human deaths worldwide, most of which occur in rural areas in developing countries [2]. One major contributing factor to this significant burden is the low availability of safe, affordable and effective biologics—in addition to rabies vaccine, timely administration of immune globulin is critical to preventing rabies by post-exposure prophylaxis (PEP). Currently, rabies immune globulins (RIG) are either prepared from human (HRIG) or equine (ERIG) plasma. Concerns such as safety, immunogenicity, sustainability, and batch-to-batch variation have led the pursuit of a replacement for RIG [3]. As a guide to design of future biologics, the WHO recommends combining monoclonal antibodies that target at least two non-overlapping epitopes on the RABV glycoprotein (G) to overcome current RIG limitations, while maintaining the breadth of cross reactivity necessary for effective protection [4].

As the most significant member, RABV belongs to the genus *Lyssavirus*, which includes at least 14 other viral species, subdivided into three phylogroups [2]. These negative-sense RNA viruses all have similar bullet-shaped morphology. The outer G protein is the sole target of all known neutralizing antibodies against lyssaviruses [5]. Intriguingly, as reported by Bakker et al, no correlation between MAb potency and affinity for G protein was found, suggesting multiple mechanisms of neutralization could exist for different immune globulins [6]. Owing to the high diversity of G protein among lyssaviruses, cross-neutralization between different phylogroups has not been observed, although a broad-spectrum antibody RVC68 was reported recently [7]. This particular human antibody was isolated from a rabies vaccinee and found to neutralize an impressive 24 isolates across 11 lyssavirus species. If endowed with high neutralization potency, such broad-spectrum antibodies could reduce the occurrence of potential escape mutants, arising through mutagenesis or gene shuffling in nature [3]. Nevertheless, as the majority of non-rabies lyssaviruses are responsible for no or very few cases of human rabies, most research in vaccine and monoclonal antibody (MAb) development has focused on RABV [2].

The antigenicity of RABV G protein has been studied thoroughly using panels of mouse MAbs over the past several decades [8–10]. Hundreds of these MAbs were analyzed for binding. Most epitopes fall within five distinct regions along the 439 amino acid ectodomain of the RABV G protein, defined as major sites I, II, III, IV, and minor site a [10, 11]. While the vast majority of murine MAbs target antigenic site II, human MAbs tend to bind antigenic sites I and III [10, 11]. For example, CL184 (CR57/CR4098) and RVC20/RVC58 are two recently developed human anti-rabies MAb cocktails that recognize antigenic site I and site III of the RABV G protein [7, 12]. The broad-spectrum protective effect exhibited by these MAb cocktails suggests the strategy of targeting two non-overlapping epitopes is highly effective. In addition, most residues within antigenic sites I and III are highly conserved among RABV variants [7]. Targeting conserved regions in RABV G is advantageous not only in ensuring broad coverage but also in helping to reduce the occurrence of escape mutants.

In the present study, we report the characterization of a novel anti-rabies MAb cocktail consisting of two newly identified humanized MAbs CTB011 and CTB012. While CTB011 targets residues at and near antigenic site III, CTB012 binds to highly discontinuous conserved residues that do not belong to any of the current antigenic sites. At equal molar ratios, the CTB011/CTB012 cocktail neutralized street RABV variants and protected against RABV-challenged Syrian hamsters in experimental PEP. The data presented herein demonstrates that CTB011/CTB012 are promising candidates for next-generation PEP of exposed humans.

## Methods

### Mouse immunization and isolation of hybridomas

Female Balb/c mice were immunized intraperitoneally (i.p.) with rabies vaccines Rabipur (Flury-LEP strain; Chiron Behring GmbH & Co., Liederbach, Germany), and Speeda (PV-2061 strain; Liaoning Chengda) mixed with complete Freund’s adjuvant. The mouse antibody titers to RABV G protein were monitored until maximal levels were achieved. Hybridomas were then generated by fusion of splenocytes with partner cells (P3X63Ag8.653 mouse myeloma cells). Hybridoma supernatants were screened for binding activity towards RABV G protein by ELISA. Based upon reactivity, clones were purified from hybridoma cultures by protein A sepharose chromatography (GE).

### Humanization and protein production of 3D11E3 and 7G11A3

Murine MAbs 3D11E3 and 7G11A3 were humanized by CDR grafting, in which human germline sequences homologous to the variable regions of 3D11E3 and 7G11A3 were chosen as the acceptor for humanization. CDR sequences from 3D11E3 were grafted into GenBank sequence DA980102 for V_H_ and CB958542 for V_L_. Similarly, CDR sequences from 3D11E3 were grafted into GenBank sequence U96282 for V_H_ and X72466 for V_L_. The final variable sequences were cloned into a mammalian expression vector carrying human gamma-1 and kappa constant regions. Humanized 3D11E3 and 7G11A3 (CTB011 and CTB012) were then produced from stably transfected CHO DG44 cells for various characterizations described herein.

### Cells

Mouse neuroblastoma (MNA) cells were grown at 37°C in 5% CO_2_ in RPMI 1640 medium (Gibco), supplemented with 10% heat-inactivated fetal bovine serum (FBS). BSR cells (a clone of baby hamster kidney-21 cells) were grown at 37°C in 5% CO_2_ in Dulbecco’s modified Eagle’s medium (Gibco) supplemented with 10% FBS and 10 mM MgCl_2_.

### Virus

Monolayers of BSR cells were infected with RABV strain CVS-11 at a multiplicity of infectivity of 0.3 for 15 min at 37°C in 0.5% CO_2_. At the end of incubation, the RABV inoculum was removed and cells were allowed to further incubate in fresh medium for 40 h at 37°C in 0.5% CO_2_. The culture supernatants were harvested and stored at -80°C.

### Western blot analysis

RABV G protein was analyzed via SDS-PAGE gel electrophoresis under both reduced and non-reduced conditions. After transferring contents of the gel to a polyvinylidene difluoride membrane, the membrane was blocked with 5% nonfat milk in TBST for 60 min. Subsequently, the membrane was incubated with MAbs 3D11E3, 3H10D3, 5A1C10, 6F11C1, and 7G11A3 at 1g/mL, washed and followed by incubation with horseradish peroxidase-conjugated goat anti-mouse secondary antibody at 1:2000. The blot was then washed and developed with the ECL system according to the manufacturer’s protocols.

### ELISA

In competitive binding studies, direct ELISA assays were carried out. Briefly, rabies vaccine (Rabipur, Chiron Behring GmbH & Co., Liederbach, Germany) was reconstituted with water and diluted fifty-fold for coating 96-well ELISA plates at 100 μL/well overnight at 4°C. The next day, the wells were washed and blocked. Unlabeled dilution series of CTB011 or CTB012 was added over the final concentration range of 0–20 μg/mL, and biotinylated CTB012 or CTB011 was added at 1 μg/mL. After 37°C incubation and washing, wells were probed with HRP-conjugated streptavidin.

In the cell-based ELISA, plates were coated with RABV-infected BSR cells. Approximately 5 x 10^4^ cells were added per well together with freshly prepared CVS-11, and plates were incubated at 37°C for 20–24 h. Cells were washed and fixed with 80% acetone the next day. Subsequent steps were the same as described above.

### Affinity determination

The binding affinities of CTB011 and CTB012 were determined by ELISA. Briefly, 96-well plates were coated with RABV-infected BSR cells. A dilution series of CTB011 or CTB012 was added over the final concentration range of 0.01024–100,000 ng/mL in duplicates. After 37°C incubation and washing with buffer, wells were probed with HRP-conjugated mouse anti-human IgG.

### Shotgun mutagenesis

DNA, encoding the full-length RABV G protein, was synthesized and sub-cloned into a mammalian high-expression vector. The RABV alanine scanning mutation library was created by substituting 504 of the 505 residues with alanine. The library was verified by sequencing and each mutant clone was validated by immunodetection of HEK293 cell surface expression with anti-RABV MAb 1C5 (Abcam). The transfected mutant library was then screened in duplicate by immunodetection for binding of CTB011 and CTB012. Reactivity was quantified for each mutant to identify point mutants that exhibited loss of binding.

### Generation of escape mutant viruses and genome analysis

Escape viruses were generated for CTB011, CTB012, and SYN023. Briefly, serial dilutions of CVS-11, ranging from 10^−2^ to 10^−7^ focus-forming units (FFU)/ml were incubated with CTB011, CTB012 and CTB011/CTB012 mixtures at 5 μg/mL or 20 μg/mL for 1 h at 37°C in 5% CO_2_. The mixtures were then added to BSR cells that had been cultured for 24 h in 48-well plates. After 3 days of incubation in the presence of either human MAb CTB011, CTB012 or SYN023, medium containing potential escape viruses was harvested from each well and frozen with liquid nitrogen until further use. Subsequently, the cells were fixed with 80% acetone for 30 min at 4°C and stained 1 h at 37°C in 5% CO_2_ with an N-fluorescein isothiocyanate (FITC)-conjugated anti-RABV antibody (kindly provided by the Military Veterinary Institute). The number of foci per well was scored by immunofluorescence, and supernatants from wells infected with the lowest dilution of RABV which produced one to six fluorescent foci were chosen for escape virus amplification. Candidate escape viruses were amplified in the presence of the respective human MAb until BSR cells were 100% infected. The viruses were harvested and analyzed. To determine genomic sequences of the escape virus, amplified virus stocks were used to inoculate a monolayer of BSR cells. Two days post-infection, the cells were harvested, and total RNA was isolated using Trizol, according to the manufacturer’s directions. Subsequently, cDNA was obtained using RevertAid First-strand cDNA synthesis kit (ThermoFisher) according to the manufacturer’s recommendations. The PCR was performed using RABV-specific primers and 2 × trans HIFI mix (Beijing Transgen Biotech Co.). The G protein gene was cloned and sequenced.

Amplified virus stocks were also used for determining the neutralization index. Serial dilution of the escape viruses were mixed with either CTB011 or CTB012 (at 5 μg/mL or 20 μg/mL). Thereafter, 5 x 10^4^ BSR cells were added per well and incubated at 37°C in 5% CO_2_ for 24 h. Following incubation, cells were washed with PBS, fixed with acetone and stained with anti-RABV FITC antibody conjugate. Viral titers were determined in focus-forming units per milliliter. The neutralization index (NI) was calculated using the formula

$$NI=log\left[FFU/ml_{titer\;without\;IgG}\right]-log\left[FFU/ml_{titer\;with\;IgG}\right]$$

An index lower than 1.0 was considered evidence of escape from neutralization by the antibody.

### *In vitro* neutralization assay

Neutralization potency of the anti-RABV antibodies was determined *in vitro* using the Rapid Fluorescent Focus Inhibition Test (RFFIT) [13]. Briefly, serial five-fold dilutions of CVS-11 or escape mutant strains were mixed with fixed quantities of CTB011, CTB012, SYN023, or HRIG (Shandong Taibang Biological Products Co. Ltd.). BSR cells were added to the mixtures at 5 x 10^4^ cells/well and samples were incubated for 24 h at 37°C in 5% CO_2_. Following incubation, cells were washed with PBS, fixed with 80% acetone and stained with FITC-conjugated anti-RABV antibody. The resulting fluorescent focus of each sample was recorded. Viral titers were determined as follows:

Virus titer (in FFU/mL) = Mean of the fluorescence focus of the last four wells × Dilution factor / virus volume of each well.

### Complement-dependent cytotoxicity

CVS-11-infected and non-infected BSR cells were plated in 96-well plates at 5000 cells per well. The MAbs CTB011, CTB012, SYN023 were diluted with DMEM and added to the cells at 0.01, 0.039, 0.156, 0.625, 2.5, 10, 40 μg/mL. Rabbit complement (Cedarlane) was dissolved with 1 mL RPMI 1640 medium and added to the cells at 50 μL per well. After a four hour incubation at 37°C, cell viabilities were measured with CellTiter-Glo luminescent cell viability assay (Promega).

### Mouse neutralization test

The neutralization breadth of SYN023 was evaluated using 15 representative variants of Chinese street RABV (S1 Fig), which were selected based upon host, timeline of prevalence and geographical areas in China.

First, the median lethal dose (LD_50_) of the 15 RABVs were determined according to the method described previously [14]. Briefly, serial dilutions of the RABV suspensions were injected into the brains of 15–20 day old Kunming mice (10 mice per dilution). Morbidity and mortality of mice were monitored for 21 days. Any ill animals were euthanized by CO_2_ inhalation and the brains removed for detection of RABV antigens using the direct fluorescent antibody (DFA) test, as described [15]. These data were used to calculate the median lethal dose (LD_50_) using the Karber formula. Next, for the mouse neutralization test, SYN023 at 1 mg/mL, HRIG at 20 IU/mL or normal saline was mixed with 0.5 mL of the 15 RABVs (200 LD_50_/25 μL dose) in equal volume, respectively. The neutralizing reaction was performed within the period of 60 ± 5 min. After incubation at 37°C for 1 h, the mixtures were injected into the brains of mice at 25μL (100 LD_50_) per mouse, 10 mice per group. The mice were monitored for 14 days after injection. Any illness or death within 4 days was treated as a non-rabies related case. The brain tissues of mice that succumbed after 4 days were collected for immunofluorescence-staining to confirm RABV infection. Animal experiments described in this section were conducted according to the Guidelines on the Humane Treatment of Laboratory Animals stipulated by the Ministry of Science and Technology of the People’s Republic of China and approved (license no. 2014–072) by the Animal Welfare Committee of the Military Veterinary Research Institute, Changchun, China.

### Modified RFFITs

The neutralization breadths of CTB011, CTB012, and SYN023 on representative North American street RABV were evaluated in a modified RFFIT assay. RABV variants were obtained from rabies diagnosis brain samples submitted to KSVDL. The variants were selected to represent the common strains found in bats and terrestrial animals in the North America. They were grown on MNA cells to sufficient titer for use in modified RFFIT procedures where the variants were used in place of the CVS-11 and the incubation time extended to 40–44 hours [13]. Briefly, the CTB011, CTB012, and SYN023 were tested at a concentration of 0.005 mg/mL against each RABV and serial five-fold dilutions were prepared. Wild-type RABV variants diluted to a theoretical (target) dose of 30–100 TCID_50_ were added to the serially diluted MAbs. The mixtures were added to 8-well lab-tek chamber slides and incubated at 37°C for 90 min to allow neutralization of viruses by the antibodies. After the incubation, 4 x 10^5^ MNA cells were added to each well for a further incubation of 40–44 h at 37°C in 0.5–2% CO_2_. At the end of the incubation, the cells were washed, fixed in 80% cold acetone, dried and stained with FITC-conjugated anti-RABV antibody (Millipore). Twenty fields/well were examined under 100x magnification using a fluorescence microscope, and the RABV neutralizing antibody (RVNA) titers (ED_50_) were calculated using the Reed and Muench method. The ED_50_ of the samples were compared to the ED_50_ of the HRIG diluted to 1.0 IU/mL to express the result in International Units (IU/mL).

### *In vivo* challenge test

The efficacy of the mouse MAb pair 3D11E3/7G11A3 and humanized MAb counterparts CTB011/CTB012 were evaluated against HRIG in Syrian hamsters Infected with RABV BD06 (a Chinese RABV isolate from a dog in Baoding area, Hebei Province) or *Tadarida brasiliensis* (a North American RABV isolate from naturally infected bats).

In RABV BD06-infected models, Syrian hamsters were first challenged with virus BD06 at 100 LD_50_ on Day -1. Animals were then vaccinated with human diploid cell vaccine (HDCV; Imovax, Sanofi Pasteur, Swiftwater, PA, USA) at 24 h after RABV challenge, and treated with 3D11E3/7G11A3 cocktail consisting of equal amounts of 3D11E3 and 7G11A3 (0.5 mg/kg) or 20 IU/kg human rabies immune globulin (Shuanglin Pharmaceutical) at 24 hour or 72 hour post RABV infection, administered at the site of virus inoculation (i.e., right gastrocnemius). Additional doses of vaccine were administered in the left gastrocnemius muscle on Days 3, 7, 14, and 28. Control groups received vaccine alone or normal saline. Hamsters were examined daily and euthanized if clinical signs of rabies infection were observed.

In a separate RABV infection model, Syrian hamsters were challenged with RABV *Tadarida brasiliensis* at 50 LD_50_/50 μL on Day -1. Prophylaxis was initiated 24 h later with vaccine (diluted with PBS; RabAvert, Novartis Vaccines, Germany) alone, vaccine plus HRIG (20 IU/kg; Imogam, Sanofi-Pasteur, Swiftwater, PA, USA), or vaccine plus 1, 0.3, 0.1, and 0.03 mg/kg of SYN023. Two control groups were included in this experiment. In addition to the vehicle control group, one group was vaccinated and treated with saline. The administration volume of all the reagents, including SYN023, HRIG and rabies vaccine, was set to 50μL per animal. The hamsters were vaccinated one dose per day on Days 0, 3, 7 and 14, while antibodies were administered once only on Day 0. Both the antibodies and RABV were injected into the right gastrocnemius muscle. The rabies vaccine was injected into the left gastrocnemius muscle. The test animals were examined two times daily for clinical signs of rabies. Once signs of illness appeared, animals were euthanized via CO_2_ inhalation and brain tissues were collected and tested for RABV virus antigens by DFA test [15]. The hamsters were maintained and evaluated at up to 35 days after infection. At the end of the experiment, all survivors were euthanized and blood was collected by cardiac puncture. The RVNA (IU/mL) of the sera were determined by RFFIT [13].

Statistical significance of survivorship differences in these animal studies was evaluated by the Log-rank test (GraphPad Prism).

### Ethics statement

The in vivo challenge studies were approved by the Institutional Animal Care and Use Committee (IACUC) for Kansas State University under Protocol # 3433. This protocol is in full compliance with the Animal Welfare Act (and its subsequent amendments) and the Health Research Extension Act of 1985. Kansas State University also filed an assurance statement (D16-00369) with the NIH Office of Laboratory Animal Welfare. The Syrian hamsters were obtained from Charles River, and the use was approved under the IACUC.

The mouse neutralization tests described in this study were conducted according to the Guidelines on the Human Treatment of Laboratory Animals stipulated by the Ministry of Science and Technology of the People's Republic of China and approved (license no. 2014–072) by the Animal Welfare Committee of the Military Veterinary Research Institute, Changchun, China. The Kunming mice used in the mouse neutralization study were obtained from Experimental Animal Center, Changchun Institute of Biological Product. The use of these animals for this study was approved by Science and Technology Agency, Jilin Province (IRB).

BSR cells and the RABV strain CVS-11 were kindly provided by Dr. Yanxing Han from the Institute of Materia Medica, Chinese Academy of Medical Sciences & Peking Union Medical College. Work associated with BSR cells and CVS-11 strain was approved by Synermore Biologics Co., Ltd. Laboratory Biosafety Management (Protocol #SYN023-PDA-SOP-001).

The MNA cells were obtained from Diagnostic Hybrids (Quidel); work with MNA cells and various rabies virus strains was approved by the Kansas State University Institutional Biosafety Committee (Protocol #991.0).

## Results

### Characterization and selection of MAbs against RABV

To generate MAbs capable of neutralizing RABV, Balb/c mice were immunized with rabies vaccines. Stable hybridomas were obtained by fusion of mouse myeloma cells with splenocytes from immunized mice. Clones were screened for G protein binding activity by ELISA. The relative binding strength, as determined by absorbance, for the top five clones are shown in Table 1. Next, standard RFFIT for neutralization were conducted for these MAbs. The 50% neutralizing titers were compared with the 50% neutralizing titer of the Chinese national HRIG standard, which was calibrated at 21.4 IU/mL. The results of the RFFIT using CVS-11 are shown in Table 1. Consistent with the findings by Bakker et al, no correlation between binding affinity and neutralizing potential was observed, indicating that sites of antibody binding play a key role in determining the neutralizing capability.

**Table 1:** *In vitro* characterization of mouse MAbs.

Clone	Relative binding	Neutralizing potency (IU/mg)	Binding epitope
**3D11E3**	++	3676	Bin 1; linear epitope
**3H10D3**	+++	3101	
**5A1C10**	++	2110	
**6F11C1**	+	4244	Bin 2; conformational epitope
**7G11A3**	+	701	

To characterize the epitope of the MAbs, purified antibodies were used as probes against non-reduced and DTT-reduced G proteins in Western blot analyses. The RABV G protein was recognized by all five antibodies under non-reducing conditions, but only 3D11E3, 3H10D3, and 5A1C10 bound reduced G protein, demonstrating the epitopes were linear. In addition, 6F11C1 and 7G11A3 exhibited significantly reduced binding when 0.1% SDS or β-mercaptoethanol was added to the ELISA buffer (S2 Fig). Taken together, these results show 6F11C1 and 7G11A3 recognize a conformational epitope, while the epitope recognized by 3D11E3, 3H10D3, and 5A1C10 was linear.

To maximize the neutralizing capability of a MAb cocktail, individual MAbs should bind non-overlapping epitopes. We determined the binding of five MAbs by a series of competition ELISAs. The results showed that the binding signal of 6F11C1 and 7G11A3 was unaffected by the addition of unlabeled 3D11E3, 3H10D3, or 5A1C10, and vice versa, showing that 6F11C1 and 7G11A3 belong to one epitope bin while 3D11E3, 3H10D3, and 5A1C10 belong to a different epitope bin (Table 1). The MAbs 7G11A3, 3D11E3, and 3H10D3 were chosen for subsequent studies, since relatively minor binding competition was observed for the 7G11A3/3D11E3 and 7G11A3/3H10D3 pairs. And among these final candidates, 3H10D3 was later eliminated due to its suppressive effect on RVNA, when administered with rabies vaccine in a non-challenged mouse neutralization test (S3 Fig). Based on all the characteristics determined for the candidate MAbs, 3D11E3 and 7G11A3 were selected for the cocktail.

**Fig 1:**
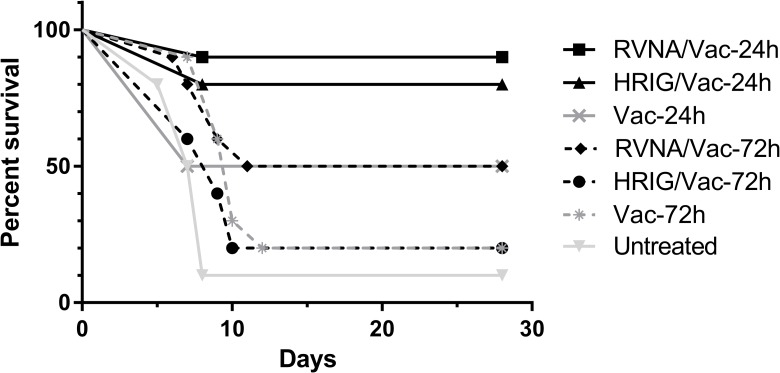
*In vivo* neutralizing activity of anti-RABV MAb cocktail compared with polyclonal HRIG. HRIG at 20 IU/kg or 3D11E3/7G11A3 cocktail (RVNA) at 0.5 mg/kg were administered in conjunction with rabies vaccine to RABV (BD06)-infected Syrian hamsters at 24 h or 72 h post infection. Vaccine was administered on Days 0, 3, 7, 14, and 28. Hamster mortality and morbidity were monitored daily. Vaccine (Vac) only and untreated groups were included as negative controls.

The *in vivo* neutralizing performance of a 3D11E3/7G11A3 cocktail was evaluated in hamster PEP models, where rabies vaccine was administered with HRIG or a 3D11E3/7G11A3 cocktail at 24 or 72 hours after infection with RABV. The mortality observed in Syrian hamsters were recorded and a survival curve was plotted, as shown in Fig 1. A survival of 90% was observed in the group treated with the 3D11E3/7G11A3 cocktail at 24 h post infection, compared to the 80% survival of the HRIG group. However, if PEP occurred 72 h post infection, survival of 3D11E3/7G11A3- and HRIG-treated hamsters dropped to 50% and 20%, respectively. These results show that 3D11E3/7G11A3 is an effective MAb pairing for PEP. In addition, the differences in survival between treatment groups were analyzed by Log-rank test and no significant difference was observed (*p*>0.05) between the 3D11E3/7G11A3- and HRIG-treated groups at both 24 h and 72 h PEP treatment.

### Complementarity between CTB011 and CTB012

**Fig 2:**
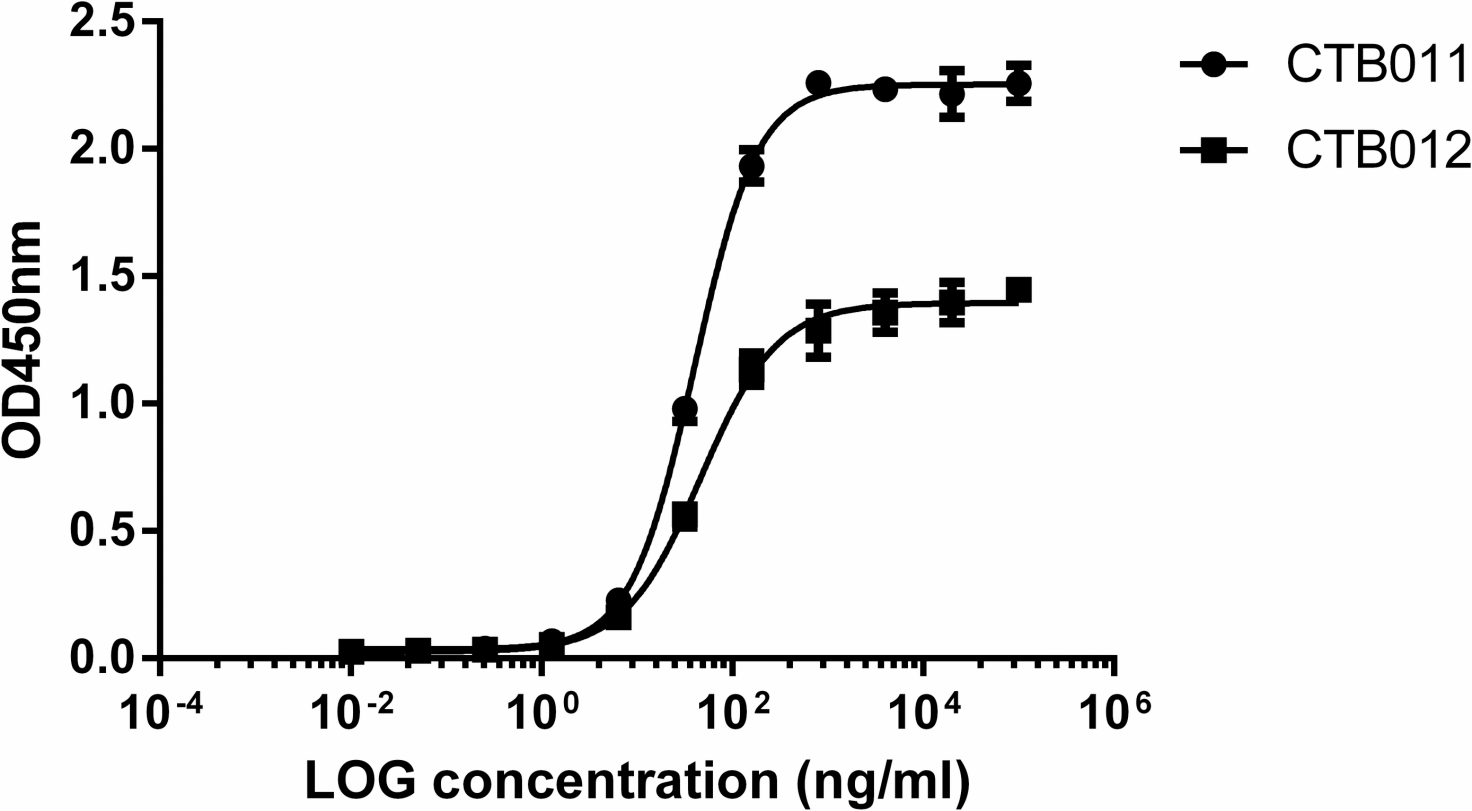
Binding affinities of CTB011 and CTB012 as measured by cell-based ELISA.

**Fig 3:**
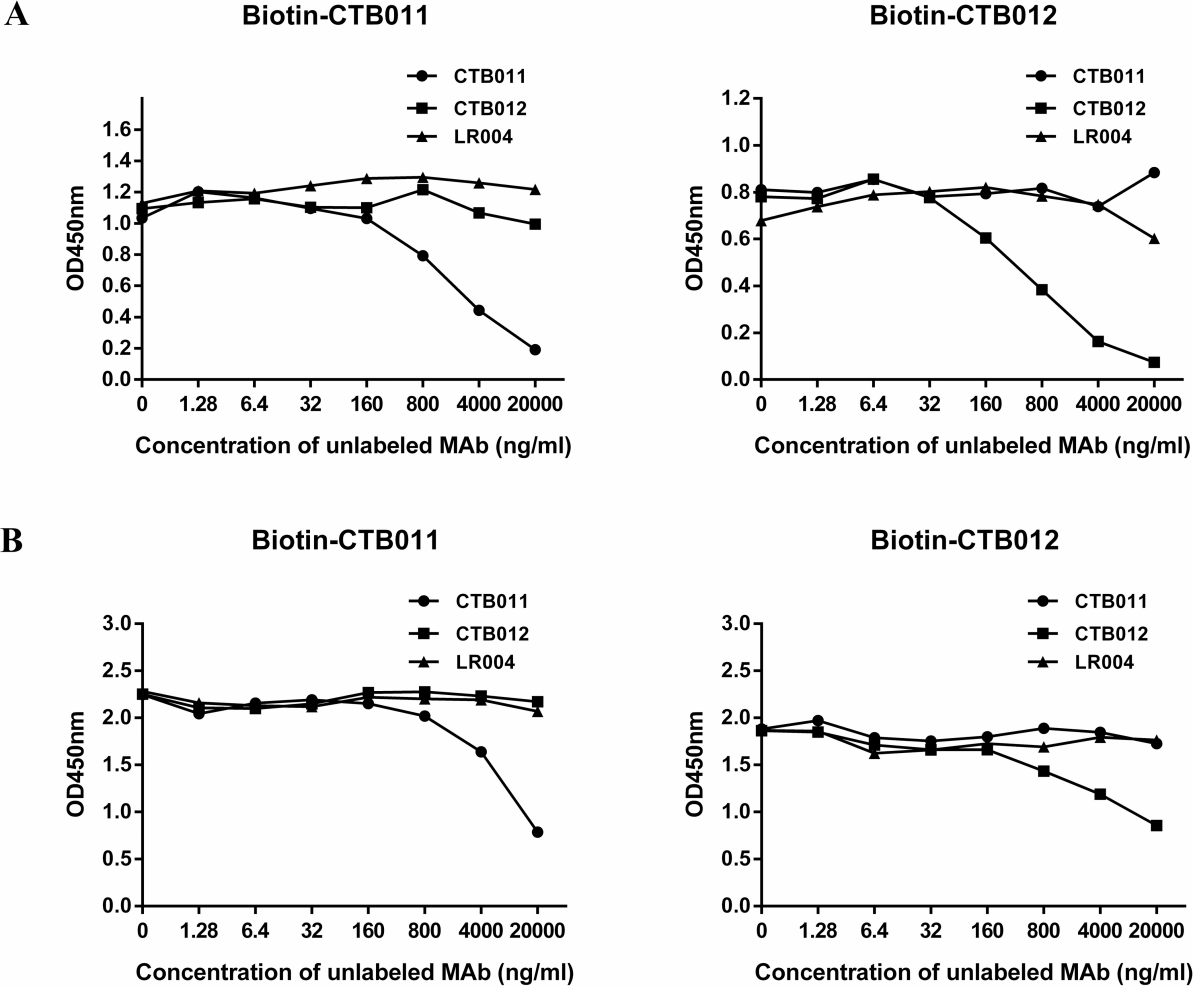
Competitive ELISA between CTB011 and CTB012. 0.1 μg of biotin-labeled CTB011 and CTB012 competed against unlabeled CTB011, CTB012, and LR004, an irrelevant antibody, over the concentration range of 0–20 μg/mL. Either diluted vaccine at 100 μL/well (A) or CVS-11 infected BSR cells (B) was coated as antigen.

To reduce immunogenicity of mouse antibodies, 3D11E3 and 7G11A, both belonging to the mouse IgG2a subclass, were humanized by CDR grafting, giving rise to MAbs CTB011 and CTB012, respectively. Human IgG1 isotype was adopted for both CTB011 and CTB012 to maximize effector functions. The neutralization potency of the four antibodies were compared in parallel by RFFIT: CTB011 maintained a higher potency in comparison to CTB012 (1300 IU/mg vs 700 IU/mg). Nevertheless, the two humanized antibodies CTB011 and CTB012 have similar affinities of 0.27 nM and 0.31 nM towards RABV G protein in a cell-based ELISA using CVS-11-infected BSR cells (Fig 2). More importantly, binding of CTB011 and CTB012 was non-competitive by ELISA. As shown in Fig 3, the binding signal of biotin-labeled CTB011 was unaffected by competition from unlabeled CTB012 up to 20 μg/mL, and vice versa. Similar results were obtained in cell-based ELISA, where the source of antigen was RABV-infected BSR cells (Fig 3B).

Based on data from competitive ELISAs, CTB011 and CTB012 interact with different regions on the RABV G protein. Next, we sought to explore the binding epitopes of CTB011 and CTB012 by generating mutant viruses that “escape” from the neutralizing effect imposed by these antibodies. The CVS-11-infected BSR cells were cultured in the presence of CTB011, CTB012, and a CTB011/CTB012 cocktail for 72 hours. The viral titers in the presence and absence (medium control) of neutralizing antibodies were determined by RFFIT and the numbers were used to calculate the neutralization index. An index lower than 1.0 was evidence of escape from neutralization by the antibody.

Sequence analyses revealed that a total of five CVS-11 mutants were generated (Table 2). First, all of the escape mutants isolated for CTB011 had a single amino acid change at position N336, denoted es011. This particular virus was neutralized by CTB012, as indicated by an index of 1.04. Similarly, escape viruses of CTB012, es012-5μg-6 and es012-20μg-5, were neutralized by CTB011 (Table 2). Es012-5μg-6 contains one single amino acid change at position 271 while es012-20μg-5 has both L271F and V272F mutations, demonstrating that both of these positions are important for CTB012 binding. The cross-neutralization of escape variants observed for CTB011 and CTB012 is consistent with the two MAbs targeting different regions on the RABV G protein. Nonetheless, if multiple mutations occur simultaneously at positions essential for CTB011 and CTB012 binding, as in the case of es011/012-1 and es011/012-4, then these mutant strains may not be neutralized by the CTB011/CTB012 cocktail. A more detailed analysis of the natural repertoire of amino acids at these positions is described in the following section. In summary, based on these experiments, residue 336 is important to CTB011 binding and positions 270–272 is involved in CTB012 binding.

**Table 2:** Summary of escape viruses generated by CTB011, CTB012, and a CTB011/CTB012 cocktail.

Escape virus	Neutralization index			Amino acid No.	Amino acid change	Codon change
	CTB011	CTB012	CTB011/CTB012			
**es011**	-0.59	1.04	ND^a^	336	N to K	AAT to AAG
**es012-5μg-6**	6.52	0.01	ND^a^	271	L to F	CTC to TTC
**es012-20μg-5**	6.84	-0.01	ND^a^	271	L to F	CTC to TTC
				**272**	V to F	GTT to TTT
**es011/012-1**	0.09	0.21	-0.01	270	H to Y	CAT to TAT
				**336**	N to K	AAT to AAG
**es011/012-4**	0.08	0.21	0.53	272	V to I	GTT to ATT
				**336**	N to K	AAT to AAG
**CVS-11**	3.78	1.33	4.09	N/A	N/A	N/A

^a^ND (not determined)

### Analyses of CTB011 and CTB012 binding epitopes

To more thoroughly map the binding epitopes of MAbs CTB011 and CTB012, a comprehensive alanine-scanning mutagenesis was conducted. First, the expression of wild-type CVS-11 RABV G protein was optimized in HEK293 cells. Then, an Ala-scan library was created by mutating every residue in the ectodomain of RABV G protein to alanine, from K1 to L505. Expression of the variants was verified by immunodetection with MAb 1C5, a commercial antibody that recognizes CVS-11. Coverage of the RABV G mutant library was 99.8%, with S447A being the only one excluded due to low expression. Next, CTB011 and CTB012 were screened at 0.25 μg/mL against the mutant library and binding was quantified for each mutant clone by immunodetection. Critical residues for each antibody were identified by comparing either the CTB011 or CTB012 binding profile to that of the control MAbs 1C5 and 1002. The mean binding signals and signal ranges (in brackets) of those residues are shown in Table 3.

**Table 3:** Critical residues for MAbs CTB011 and CTB012.

Residue	Mutation	CTB011	CTB012	Abcam 1C5	Abcam 1002
**2**	F2A	86.2 (3)	**1.7 (1)**	67.1 (17)	52.2 (3)
**25**	P25A	69.6 (1)	**20.9 (5)**	65.58 (22)	69.6 (1)
**26**	N26A	96.2 (3)	**4.4 (2)**	58.0 (1)	60.9 (28)
**171**	T171A	58.5 (3)	**24.8 (6)**	79.4 (48)	59.49 (3)
**281**	E281A	113.4 (30)	**17.3 (3)**	54.5 (10)	77.3 (14)
**336**	N336A	**11.3 (1)**	79.6 (0)	93.9 (38)	64.8 (9)
**339**	I339A	**6.4 (1)**	20.6 (8)	69.7 (47)	47.0 (14)
Secondary critical residues for CTB011					
**337**	E337A	**31.7 (0)**	32.3 (6)	61.8 (23)	47.7 (16)
**370**	H370A	**36.7 (6)**	127.8 (76)	100.5 (11)	124.6 (2)

The MAb CTB011 exhibited low binding to four mutant clones, N336A, I339A, E337A, and H370A, suggesting it binds a surface patch centered at position I339. This result is consistent with previous findings that the epitope is linear (Table 1), as well as the point mutation identified in the escape virus analysis (Table 2). Two of the residues, N336 and E337, fall into the short stretch designated as antigenic site III, which is also targeted by three other well-characterized MAbs: CR4098, RAB1/17C7, and RVC58 [6, 7, 16]. According to the degree of conservation analysis by Benedictis et al, residues 336 and 337 are 90.6% and 99.6% conserved among >2,500 RABV isolates [7]. Residue 339 is also conserved based on a smaller scale alignment of 67 lyssavirus G protein sequences, yet no known antibody has been demonstrated to bind at position 339. Based upon these data, the epitope of MAb CTB011 is unique and relatively conserved.

In contrast to MAb CTB011, the critical residues for CTB012 binding were not as distinct. Under the same conditions, binding of CTB012 to the entire mutant library was of weaker intensity in comparison to CTB011. Through careful examination of the CTB012 binding profile, mutants F2A, P25A, N26A, T171A, and E281A most consistently displayed low binding towards CTB012 in comparison to CTB011, MAbs 1C5 and 1002. These residues are rather dispersed throughout the G protein and they do not belong to known antigenic sites, suggesting a completely novel binding site for MAb CTB012. Based on the most homologous G protein crystal structure available for another rhabdovirus (PDB# 2J6J; vesicular stomatitis virus), P25, N26, and E281 are clearly closer in space, whereas residues F2 and T171 are further away from the patch formed by residues 25, 26, and 281. Considering that RABV G protein undergoes conformational changes with at least three configurations [17], these could be positioned in closer proximity with one another under certain conditions.

Positions 270–272 were also identified in a second experiment where the mutant library was screened with CTB012 Fab. These residues coincide with the critical residues identified in the escape mutant study. One reason these mutants did not stand out in the first experiment may be that the mutations in the escape viruses, such as H270Y and L271F, are much more drastic changes than mutating to alanine. Apparently, alanine mutations at residues 270–272 have less impact on CTB012 binding compared to the aforementioned five residues. Further research would be required to gain a more comprehensive view of the binding epitope of mAb CTB012.

Despite the highly conformational nature of the CTB012 epitope, all of the residues currently implicated in CTB012 binding were highly conserved across RABV and non-rabies lyssaviruses (Table 4). In the G protein sequence alignment of 83 RABV isolates, F2, P25, N26, T171, E281, or even H270–V272 are completely conserved (100%). By contrast, more variations in the H270–V272 region are seen across the non-rabies lyssaviruses. Among the 130 lyssaviruses analyzed, H270, L271, V272 were 97.7, 94.7, and 76.6% conserved respectively, which is in great contrast to the five residues identified through alanine scanning. A high degree of conservation of the binding epitope could be advantageous in determining the breadth of neutralization for CTB012.

**Table 4:** Conservation of key residues in the binding epitope of MAb CTB012.

Viruses^a^	Positions							
	2	25	26	171	270	271	272	281
CVS-11	F	P	N	T	H	L	V	E
**83 RABVs**	100%	100%	100%	100%	100%	100%	100%	100%
**47 non-rabies lyssaviruses**	100%	100%	98% (2% D)	100%	94% (4% N 2% E)	86% (4% M 4% F 4% A 2% S)	38% (62% I)	100%
**Overall**	**100%**	**100%**	**99.2%** (0.8% D)	**100%**	**97.7%** (1.5% N 0.8% E)	**94.7%** (1.5% M 1.5% F 1.5% A 0.8% S)	**76.6%** (23.3% I)	**100%**

^a^A list of the strains used in this analysis is included in S1 Table.

### Complement-dependent cytotoxicity of CTB011, CTB012, and SYN023, a CTB011/CTB012 cocktail

**Fig 4:**
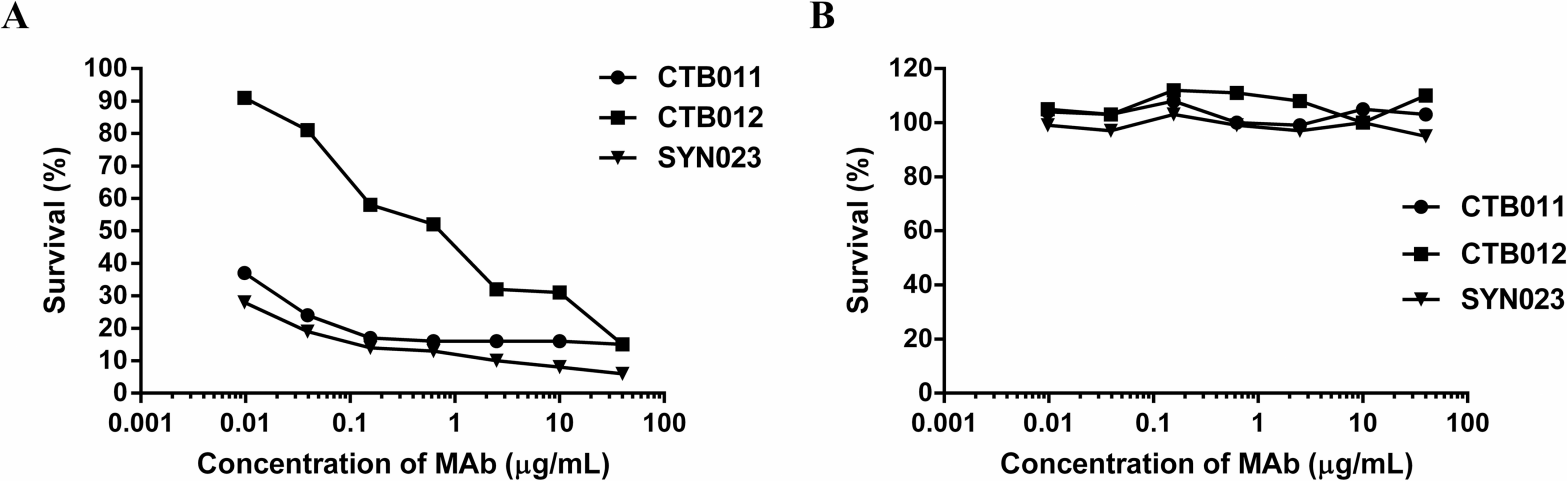
Complement-dependent cytotoxicity of MAbs CTB011, CTB012, and SYN023. CVS-11 infected BSR cells (A) and non-infected BSR cells (B).

In addition to being able to bind and neutralize RABV, the Fc portion of antiviral antibodies often contribute to viral clearance by mediating functions such as antibody-dependent cellular cytotoxicity (ADCC) and complement-dependent cytotoxicity (CDC). To assess if mAbs CTB011 and CTB012 are able to mediate ADCC, standard ADCC assays were carried out where freshly prepared PBMC effector cells were incubated with CVS-infected BSR cells in the presence of MAbs CTB011, CTB012, SYN023, or HRIG. The results showed that no significant ADCC was observed for any of the immune globulins (S4 Fig). Similarly, an *in vitro* model based on BSR cells was established to detect CDC activity. In this assay, CVS-11 infected and non-infected BSR cells were incubated with varying concentrations of MAbs CTB011, CTB012, or SYN023 in the presence of rabbit complement. Cell viability was then determined in four hours by measuring ATP levels. As shown in Fig 4A, cytotoxicity was observed for virus-infected BSR cells that had been incubated with CTB011, CTB012, and SYN023 in a dose-dependent fashion. By contrast, no toxicity was observed for non-infected BSR cells over the entire antibody concentration range, showing that cytotoxicity was specifically associated with G protein-expressing cells, which are targeted by CTB011 and CTB012 (Fig 4B). These data suggest that CTB011 and CTB012 are capable of mediating CDC as a mechanism of clearing RABV.

### Breadth of neutralizing activity of CTB011, CTB012, and SYN023

The potency and breadth of neutralization are two of the most crucial parameters in determining the value of an antiviral biologic. To evaluate the neutralizing power of CTB011 and CTB012, the optimal ratio of the two MAbs was determined by RFFIT (S2 Table). Higher proportions of CTB011 (2:1 and 3:1) were associated with slightly better potency against CVS-11 compared with CTB012 (1:2 and 1:3). Considering the high degree of conservation of the CTB012 epitope, a 1:1 ratio was adopted and designated as SYN023. The potency of CTB011, CTB012, and SYN023 were determined to be 1300±200, 740±50, and 1700±300 IU/mg, respectively.

To gain a perspective on the neutralization breadth of SYN023, its neutralizing effects against additional RABVs were determined in parallel to HRIG. As shown in Table 5, both SYN023 and HRIG were able to provide protection against all of the RABVs. This result is in agreement with G protein sequence analysis of the 15 viruses, in which all of the critical residues for CTB011 and CTB012 were found conserved. Although 10–30% mortality rates were associated with ZJ-QZ, JX08-45, JX13-235, and JX10-37 in this experiment, in a separate experiment, either CTB011 or CTB012 alone showed complete neutralization (100% survival) against three of the isolates, ZJ-QZ, JX13-235, and JX10-37 at 0.3 mg/mL. Based on this experiment, SYN023 was able to protect against prevalent Chinese street RABVs.

**Table 5:** Mouse neutralization test on SYN023.

Virus	Type	SYN023	HRIG
**HN10**	Street RABV	+	+
**Hubei**	Street RABV	+	+
**ZJ-QZ**	Street RABV	+^a^	+
**Shanxi-HZ-6**	Street RABV	+	+
**BD06**	Street RABV	+	+
**JX13-189**	Street RABV	+	+
**JX08-45**	Street RABV	+^a^	+
**JX13-235**	Street RABV	+^a^	+
**JX12-234**	Street RABV	+	+
**JX09-17**	Street RABV	+	+
**JX13-417**	Street RABV	+	+
**JX10-37**	Street RABV	+^a^	+
**JX13-228**	Street RABV	+	+
**ZJ12-03**	Street RABV	+	+
**ZJ13-431**	Street RABV	+	+

^a^These strains displayed 70–90% survival rates depending on experimental setup.

The neutralizing capability of SYN023 against common North American RABVs was also explored in a modified RFFIT using MNA cells. Both SYN023 and HRIG were able to neutralize all 10 of the wildlife RABVs (Table 6). Interestingly, CTB011 and CTB012 each neutralized 7 and 10 strains independently. Owing to the high degree of conservation of the binding epitope, CTB012 was able to neutralize 3 isolates that were not neutralized by CTB011. Moreover, CTB012 was highly potent against 5 RABVs—North Central Skunk, South Central Skunk, Texas Grey Fox, red bat (*Lasiurus borealis)*, and big brown bat (*Eptesicus fuscus*), thereby contributing significantly to the overall potency of SYN023 against these isolates. To summarize, SYN023 was found to have a broad neutralization spectrum, with neutralizing effects equivalent or superior to HRIG against 15 Chinese RABVs and 10 North American wildlife isolates.

**Table 6:** SYN023 neutralization activity against North American wildlife RABV isolates.

Virus	CTB011	CTB012	SYN023	HRIG
***CVS-11***	+	+	+	+
***North Central Skunk***	+	+	+	+
***Texas Grey Fox***	+	+	+	+
***Lasiurus borealis***	+	+	+	+
***Eptesicus fuscus***	+	+	+	+
***South Central Skunk***	+	+	+	+
***Florida Raccoon***	-	+	+	+
***Lasiurus cinereous***	+	+	+	+
***SW Eptesicus fuscus***	-	+	+	+
***Tadarida brasiliensis***	-	+	+	+
***Eastern Tri-colored***	+	+	+	+

### *In vivo* post-exposure prophylaxis protection by SYN023

**Fig 5:**
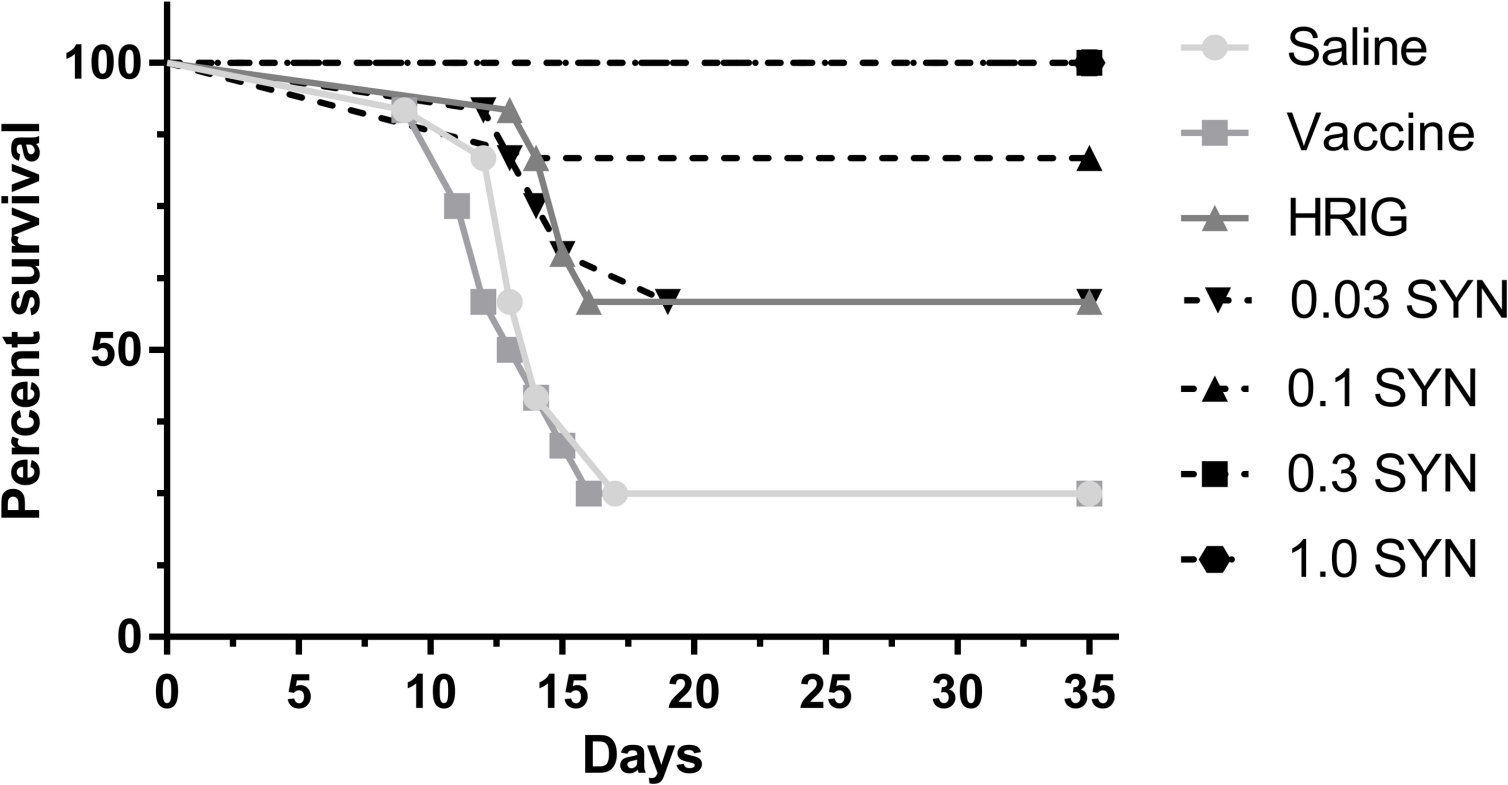
Rabies immune globulin post-exposure protection in a Syrian hamster model. Groups of Syrian hamsters were injected with 50 LD_50_ of a bat (*Tadarida brasiliensis*) RABV. At 24 hours post-infection, SYN023 at 0.03, 0.1, 0.3, 1.0 mg/kg and HRIG at 20 IU/mL was injected along with anti-rabies vaccine into the same sites as infection. Additional doses of vaccines were administered on Days 3, 7, and 14. The morbidity and mortality of the hamsters were monitored for up to 35 days post-infection. Numbers of surviving hamsters for each group are plotted in this graph by day. Vaccine only and saline treated groups were included as negative controls.

As demonstration of *in vitro* neutralizing capacity of an antibody does not necessarily warrant efficacy in post-exposure models [18], the post-exposure neutralizing capability of SYN023 was evaluated against HRIG in Syrian hamster models. The SYN023 at 0.03, 0.1, 0.3, 1.0 mg/kg and HRIG at 20 IU/mL was injected along with anti-rabies vaccine into the right leg of Syrian hamsters at the same site where 50 LD_50_ of RABV (*Tadarida brasiliensis*) was administered to infect the hamsters 24 hours prior. As shown in Fig 5, only 25% of the animals survived in the saline control group as well as in the vaccine only group. The HRIG-treated hamsters had a 58% survival rate up to 35 days after RABV infection. Alternatively, a clear dose-dependence effect was observed for SYN023 as the survival rates were 58, 83, 100, and 100% over the concentration range from 0.03 to 1.0 mg/kg. Similar survivorship was achieved for the lowest dose level test for SYN023 at 0.03 mg/kg and HRIG-treated at current dosage level (*p*>0.05 by Log-rank test), demonstrating the potency of SYN023 in these PEP models.

## Discussion

Rabies is an ancient zoonotic disease, known for thousands of years [19, 20]. Despite the development of rabies vaccines for over a century, rabies continues to impose a major burden upon public health, especially in the developing world [1]. In fact, half of the world’s population live in areas where rabies is enzootic [4, 21]. Annually, among the 15 million people who receive some form of PEP, less than 10% of patients with category III exposures receive RIG due to high cost and low availability [3]. Clearly, RIG is indispensable in PEP. Apart from cost and supply, batch-to-batch variation and risks related to the use of blood-derived products are also issues that urgently need to be addressed [2]. One well-recognized alternative to current RIG would be an anti-rabies MAb cocktail [22]. Ideally, a cocktail, consisting of at least two non-competing MAb that specifically target highly conserved G protein regions, has the best chance at tackling a broad spectrum of domestic and wildlife and street RABVs [2, 23]. Here, we report the development and characterization of SYN023, a 1:1 mixture of two novel anti-rabies MAbs, CTB011 and CTB012, that fulfills such criteria. In multiple comparative studies against HRIG, SYN023 had similar neutralization potency and breadth of protection as HRIG, demonstrating its potential as effective rabies biologic in PEP.

The RABV G protein has been the subject of intense investigation not only for its role in host cell attachment and membrane fusion [5], but also as an important antigen for inducing protective immunity [3]. Antibodies with high affinities towards RABV G protein neutralize by physically blocking the initial steps of cellular entry [24, 25]. However, in addition to strong binding, location of the binding epitope is crucial in determining the neutralizing potency of an antibody [26]. As revealed by several mechanistic studies, the number of bound IgG molecules required to neutralize a RABV virion could vary from 20 to over 1000 depending on where the epitope resides [25, 26]. This is in agreement with the finding by Bakker et al. that neutralizing potency did not correlate with binding affinity among a pool of 17 anti-RABV antibodies [6]. Thus, differences in epitope structure and location would explain the disparate neutralizing potencies displayed by CTB011 and CTB012 (Table 1; 1300 IU/mg and 700 IU/mg after humanization), given that they possess essentially the same binding affinity of around 0.3 nM towards RABV G protein.

Through escape mutant analysis and alanine-scanning mutagenesis, MAbs CTB011 and CTB012 interacted with distinct regions of RABV G protein. CTB011 binds at the edge of antigenic site III, which was defined in the early works by Flamand and co-workers to span residues 330–338 [27]. While a N336K-resistant mutant was identified for CTB011, data from shotgun mutagenesis suggests that N336 and I339 are both critical residues in the binding interface (Table 3). Several other anti-RABV MAbs currently under development, including CR4098, 17C7, and RVC58, also target antigenic site III [7, 16, 28]. Coincidently, both CR4098 and 17C7 gave rise to resistant viruses carrying the N336D mutation, and both MAbs had nearly identical potencies of around 1100 IU/mg as measured by RFFIT [6, 16]. By contrast, RVC58, whose epitope overlaps only partially with those of CR4098 and 17C7, was able to neutralize a wider selection of non-rabies lyssavirus strains in phylogroup I [7]. As evident in the sequence conservation analysis by Benedictis et al, the breadth of neutralization is largely dictated by the degree of epitope conservation, which could vary from 77% to >99% for different positions within antigenic site III. For MAb CTB011, N336 is polymorphic with 90.6% conservation among 2566 RABV while position 339 is 74.4% I and 25.5% L among 130 lyssaviruses analyzed. The relatively low degree of conservation at these two positions is reflected in the narrower breadth of neutralization of CTB011 (Table 6).

In contrast to CTB011, CTB012 interacts with several highly conserved residues novel to the current antigenic sites. Escape mutants of CTB012 had mutations at positions 270–272, closest to antigenic site IV or G5 (aa 261–264) and further away from antigenic sites II and III spatially [11]. H270, L271, and V272 are conserved 100% among 83 RABVs analyzed, 94%, 86%, and 38% conserved among 47 non-RABV lyssavirus species, giving an overall conservation of 97.7%, 94.7%, and 76.6% among 130 lyssaviruses we selected to represent 12 lyssavirus species (Table 4). However, additional residues critical to binding were identified through alanine-scanning mutagenesis: F2, P25, N26, T171, E281, with F2A and N26A near completely abolishing the binding activity of CTB012 (Table 3). Although it is difficult to decipher which residues are more important to the binding of MAb CTB012 without a crystalline structure, we did find alanine-scanning mutagenesis to be a more direct and unbiased approach for epitope mapping than escape mutant analysis, since the generation of escape viruses is complicated by other factors, such as relative pathogenicity of mutants and rates of mutation. Another possibility is that the mutations generated in the escape viruses, namely H270Y, L271F, V272F, are all aromatic ring substitutions that could bring about more drastic conformational changes than alanine mutations. Nonetheless, the five critical residues were identified with an unprecedented high degree of conservation—F2, P25, T171, and E281 were all found to be 100% conserved while N26 was 99.2% conserved among 130 lyssaviruses analyzed, providing the basis for the broad neutralizing capabilities exhibited by CTB012 against domestic animal and wildlife RABVs (Table 6).

The residues implicated in CTB012 binding are not all clustered together, based on the vesicular stomatitis virus glycoprotein G structure (PDB# 2J6J/512S), commonly used to model RABV G protein. In fact, the patch formed by positions 25, 26, and 281 seems to be close to the inter-subunit interface for trimer formation. Positions 270 to 272 are within close proximity of the patch with greater solvent accessibility. On the contrary, positions 2 and 171 are distant from the other positions potentially involved in CTB012 binding, suggesting that some of the mutations employed in our studies could have had an indirect impact on binding. Further research will help to refine the binding epitope of CTB012.

The CTB012 (7G11A3) was originally selected from a pool of anti-RABV hybridoma clones for its complementarity with CTB011 (3D11E3). The non-competitive nature of the two MAbs was demonstrated in ELISAs (Fig 3) and in the cross-protection from escape mutants (Table 2). In addition, CTB012 was able to neutralize 3 viruses that were not recognized by CTB011, presumably due to its unique epitope. When combined at equal molar ratios, the neutralizing power of each MAb was fully maintained in the anti-rabies cocktail SYN023, allowing the neutralization of 10 North American street RABVs *in vitro* and 15 prevalent Chinese RABVs in animal models. Not only did SYN023 exhibit the same breadth of neutralization as HRIG, but also displayed a significant higher potency against half of the North American wildlife RABVs examined (Table 6). Based on current data, SYN023 has shown sufficient breadth of protection against relevant RABVs, and is expected to be an effective replacement of HRIG.

Like any other RNA virus, RABV is evolving in nature and undergoing antigenic drift among diverse hosts such as bats [29]. Although the majority of non-rabies lyssaviruses have not been associated with human deaths, all are fully capable of causing fatal neurological diseases in animals and humans [30]. Sporadic cases of human rabies have been reported for EBLV1, EBLV2, ABLV, IRKV, DUVV, and MOKV over the years [2, 31]. Current RIG products have limited activity against non-rabies lyssaviruses [31]. Commercial HRIG lacked neutralization capability against MOKV, LBV, EBV1 [32, 33], WCBV [33], and DUVV. Similarly, two of the antibody-based anti-rabies MAbs currently in clinical development, CL184 and RAB1, were unable to neutralize most non-RABV lyssavirus species across the phylogroups [7]. By contrast, CTB012 is predicted to neutralize many lyssaviruses based on sequence analysis (Tables 4 and 6). As such, a comprehensive characterization with respect to all lyssavirus species is underway to help determine the full neutralization scope of SYN023.

In the event of a productive viral infection, virus-specific antibodies play essential roles in the neutralization and clearance of such pathogens. Effective antiviral antibodies may even participate in the removal of virus-infected cells by mediating ADCC or CDC, alone or in tandem [34, 35]. For example, CDC has been demonstrated for several neutralizing antibodies in the clearance of West Nile and influenza A viruses [36]. Here, we show that CTB011 and CTB012 are both able to specifically target and mediate lysis of CVS-11 infected cells in the presence of rabbit complement (Fig 4). Thus, RABV-neutralizing MAbs CTB011 and CTB012 could exhibit enhanced infectivity-inhibiting activities *in vivo* through the complement system. The efficacy of SYN023 in experimental PEP was demonstrated in bat RABV-challenged Syrian hamster models (Fig 5). In the *in vitro* neutralization assays, the *Tadarida* virus was modestly neutralized by SYN023 (Table 6). Yet, SYN023 at 0.03 mg/kg provided similar protection as 20 IU/kg of HRIG, giving an overall survival rate of 58.3% (7/12) at Day 35. Dose-dependence was observed as 0.1, 0.3, and 1.0 mg/kg of SYN023 administration protected 83%, 100%, 100% of the mice respectively. Although some interference with rabies vaccine was noted for SYN023 at a dosage level of 0.3 mg/kg, in a separate study with non-challenged Syrian hamsters, the levels of RVNA were well above the arbitrary ideal threshold level of 0.5 IU/ml at all time points up to 42 days. The optimal dose for future research will be determined based on a fine balance between potency and interference. With the strong neutralizing potency against a broad selection of RABVs, the SYN023 MAb cocktail offers a safe and cost-effective alternative for rabies PEP. Finally, we envision that reduced production cost and steady supply of RIG alternatives such as the SYN023 MAb cocktail would bolster the use of rabies PEP, particularly in endemic areas.

## Supporting information

S1 Fig Distribution of 15 representative isolates of RABV (●) in the Chinese phylogenetic tree. (TIF)

S2 Fig Binding capacity of 6F11C1 (A) and 7G11A3 (B) to rabies virus glycoprotein treated with different reagents.
(TIF)

S3 Fig Serum rabies virus neutralizing antibody (RVNA) titers in non-challenged BALB/c mice. The mice in each treatment group (n = 6 per group) were vaccinated with rabies vaccine and treated on Day 0 with 50 μg/dose 7G11A3 (A), 3D11E3 (B), 3H10D3 (C). Blood was collected from mice on Days 1, 3, 7, 14, and 28; RVNA titers were determined by rapid fluorescent focus inhibition test. The geometric mean of the titers were calculated and plotted against time. The long lines represent means and the short lines represent max and mins.
(TIF)

S4 Fig Antibody-dependent cell-mediated cytotoxicity (ADCC) of CTB011, CTB012, and SYN023 in CVS-11 infected BSR cells (A) and non-infected BSR cells (B).
(TIF)

S1 Table Degree of conservation at the CTB012 binding epitope. (DOCX)

S2 Table Neutralizing potency of CTB011, CTB012, and CTB011/CTB012 cocktails determined by RFFIT in different laboratories. (DOCX)
