# Supplementary figures and images for: SYN023, a novel humanized monoclonal antibody cocktail, for post-exposure prophylaxis of rabies
Source: PLoS Negl Trop Dis. 2017 Dec 20;11(12):e0006133. doi: 10.1371/journal.pntd.0006133 (PMC5754141; doi:10.1371/journal.pntd.0006133)

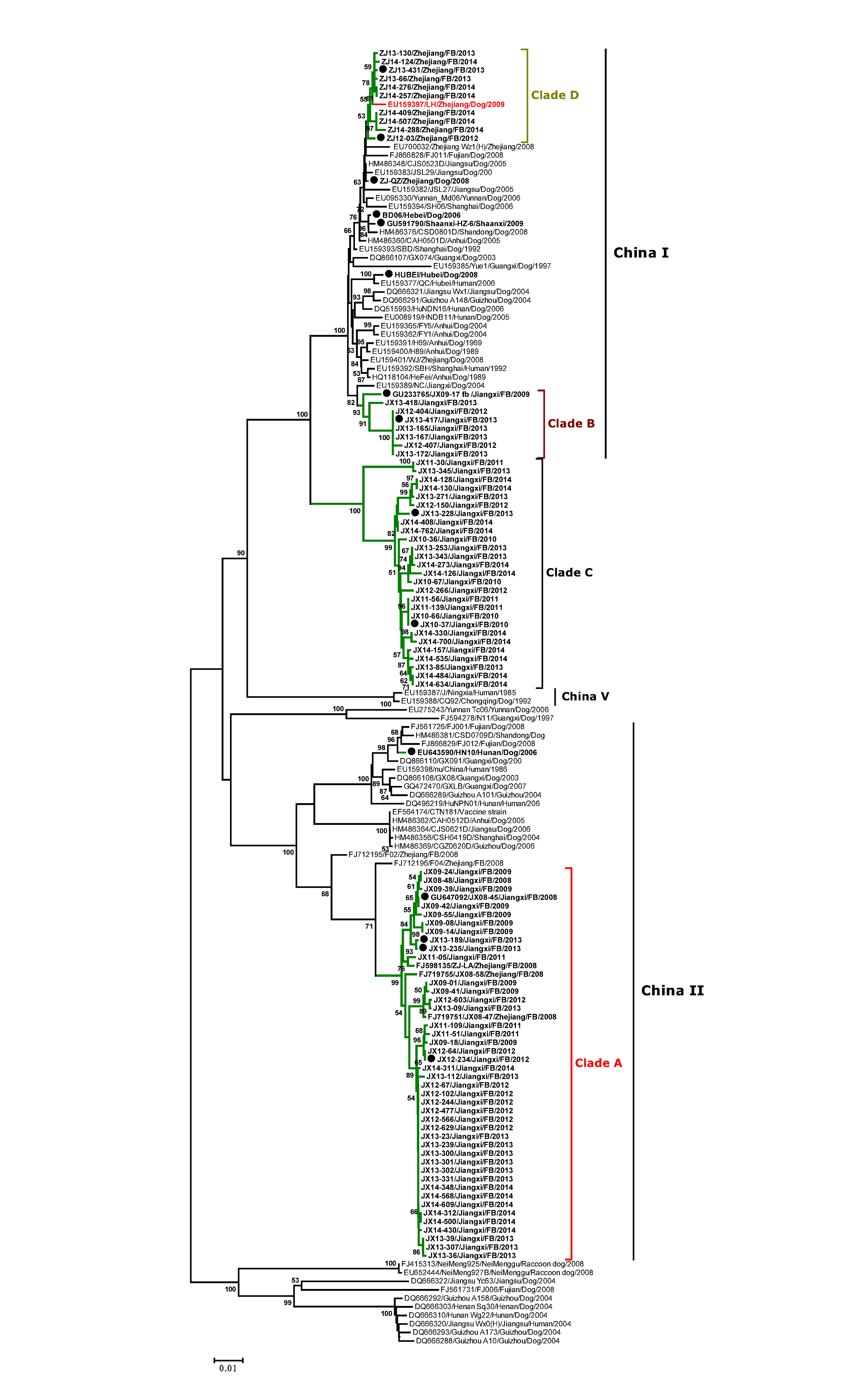

Supplement: S1 Fig — (TIF) [file pntd.0006133.s001.tif]

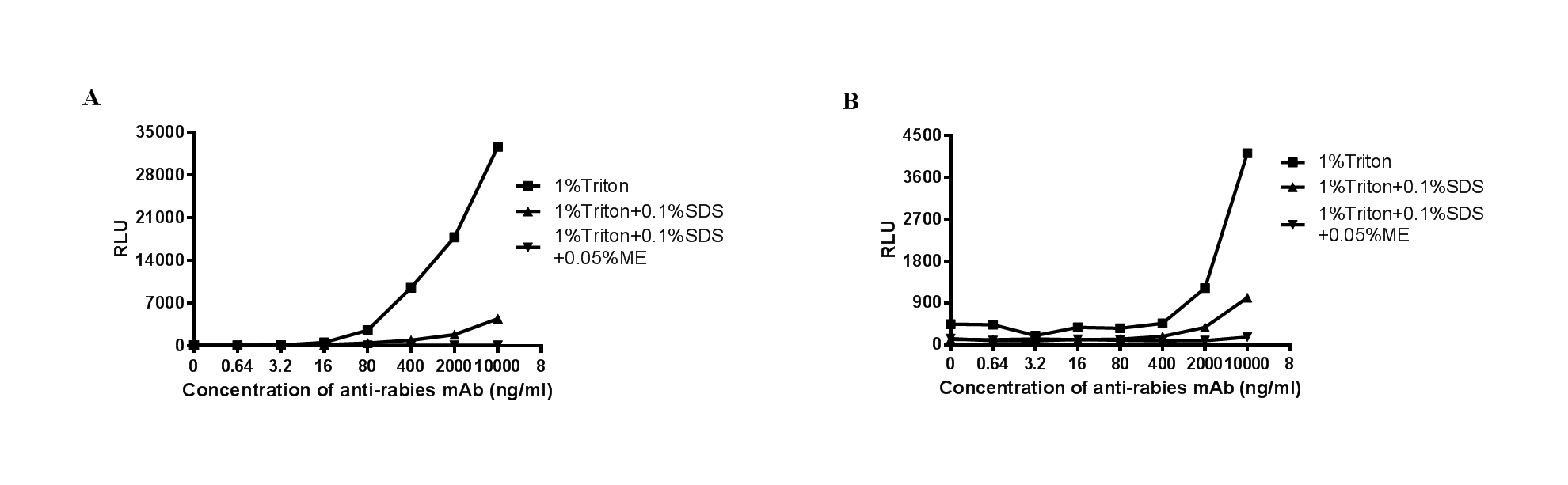

Supplement: S2 Fig — Binding capacity of 6F11C1 (A) and 7G11A3 (B) to rabies virus glycoprotein treated with different reagents. (TIF) [file pntd.0006133.s002.tif]

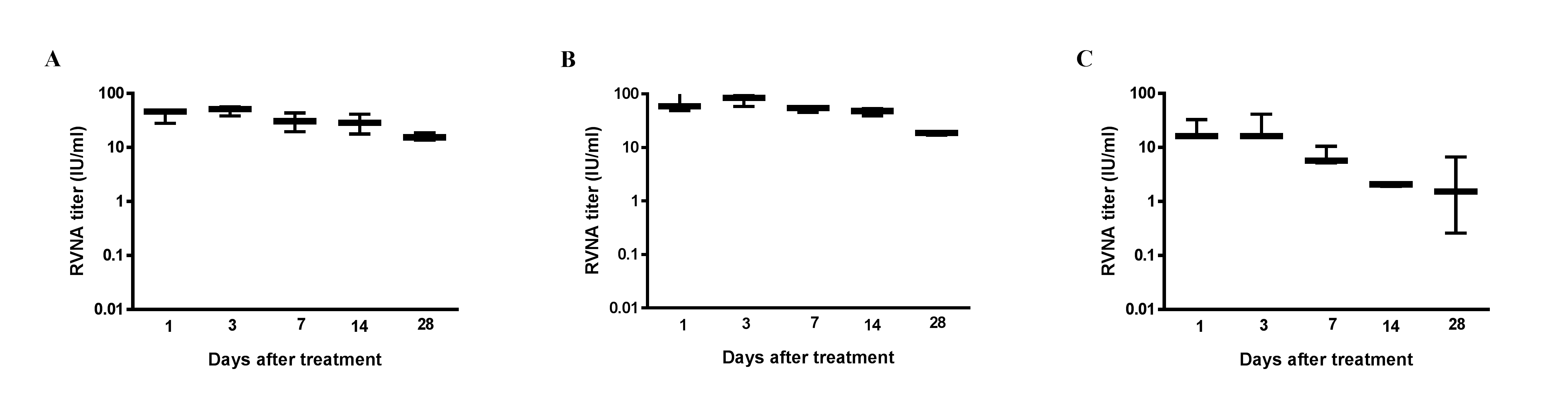

Supplement: S3 Fig — The mice in each treatment group (n = 6 per group) were vaccinated with rabies vaccine and treated on Day 0 with 50 μg/dose 7G11A3 (A), 3D11E3 (B), 3H10D3 (C). Blood was collected from mice on Days 1, 3, 7, 14, and 28; RVNA titers were determined by rapid fluorescent focus inhibition test. The geometric mean of the titers were calculated and plotted against time. The long lines represent means and the short lines represent max and mins. (TIF) [file pntd.0006133.s003.tif]

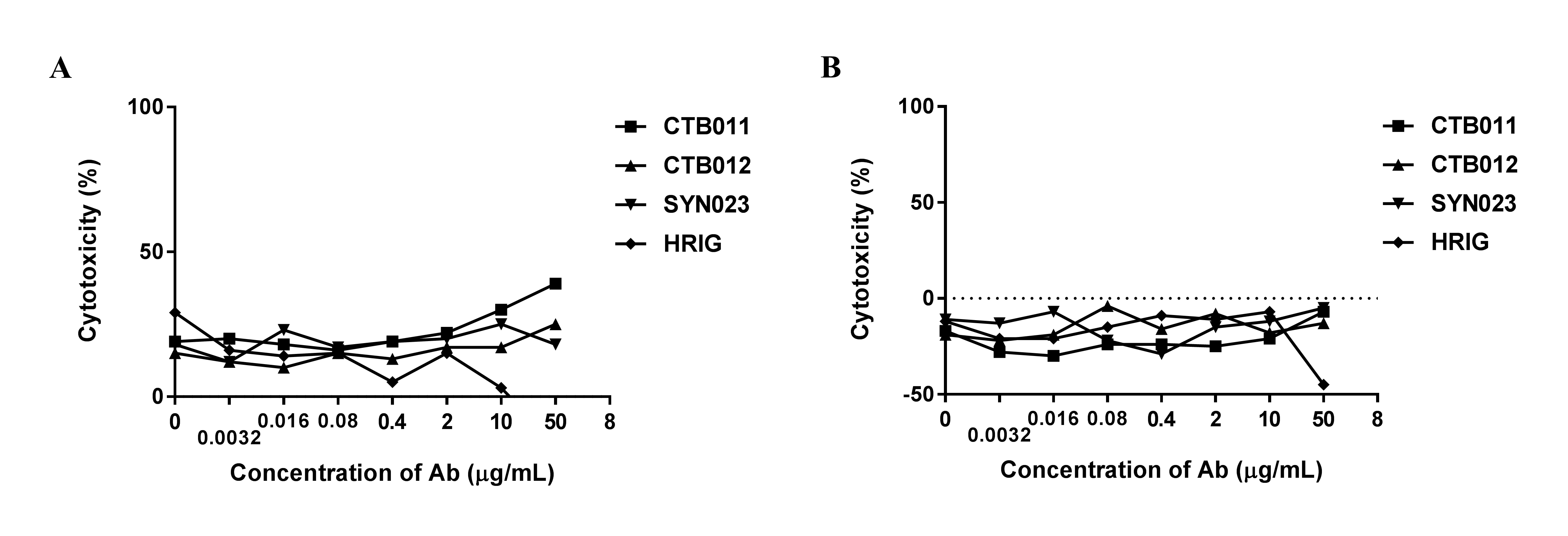

Supplement: S4 Fig — Antibody-dependent cell-mediated cytotoxicity (ADCC) of CTB011, CTB012, and SYN023 in CVS-11 infected BSR cells (A) and non-infected BSR cells (B). (TIF) [file pntd.0006133.s004.tif]
