# Supplementary material for: SYN023, a novel humanized monoclonal antibody cocktail, for post-exposure prophylaxis of rabies
Source: PLoS Negl Trop Dis. 2017 Dec 20;11(12):e0006133. doi: 10.1371/journal.pntd.0006133 (PMC5754141; doi:10.1371/journal.pntd.0006133)
Supplement: S1 Table — (DOCX) [file pntd.0006133.s005.docx]

**S1 Table. Degree of conservation at the CTB012 binding epitope.**

| Strain | Accession number | Positions | | | | |
| --- | --- | --- | --- | --- | --- | --- |
|  |  | 2 | 25–26 | 171 | 270–272 | 281 |
| RABV | CVS-11 | KFP | SCPNNI | STN | IEHLVVE | REE |
| RABV | ABZ81200 | F | PN | T | HLV | E |
| RABV | ABZ81185 | F | PN | T | HLV | E |
| RABV | AGE31951 | F | PN | T | HLV | E |
| RABV | ABN11309 | F | PN | T | HLV | E |
| RABV | ABX46661 | F | PN | T | HLV | E |
| RABV | ABX46664 | F | PN | T | HLV | E |
| RABV | ABX46649 | F | PN | T | HLV | E |
| RABV | AJ871962 | F | PN | T | HLV | E |
| RABV | BAN14123 | F | PN | T | HLV | E |
| RABV | EF206707 | F | PN | T | HLV | E |
| RABV | EU352767 | F | PN | T | HLV | E |
| RABV | KC197967 | F | PN | T | HLV | E |
| RABV | KU739036 | F | PN | T | HLV | E |
| RABV | KU739037 | F | PN | T | HLV | E |
| RABV | KU739038 | F | PN | T | HLV | E |
| RABV | KU739039 | F | PN | T | HLV | E |
| RABV | KU739040 | F | PN | T | HLV | E |
| RABV | KU739041 | F | PN | T | HLV | E |
| RABV | KU739042 | F | PN | T | HLV | E |
| RABV | KU739043 | F | PN | T | HLV | E |
| RABV | KU739044 | F | PN | T | HLV | E |
| RABV | KU739045 | F | PN | T | HLV | E |
| RABV | KU739046 | F | PN | T | HLV | E |
| RABV | KU739047 | F | PN | T | HLV | E |
| RABV | KU739048 | F | PN | T | HLV | E |
| RABV | KU739049 | F | PN | T | HLV | E |
| RABV | KU739050 | F | PN | T | HLV | E |
| RABV | KU884973 | F | PN | T | HLV | E |
| RABV | KU888637 | F | PN | T | HLV | E |
| RABV | KU888638 | F | PN | T | HLV | E |
| RABV | KU888639 | F | PN | T | HLV | E |
| RABV | KU888640 | F | PN | T | HLV | E |
| RABV | KU888641 | F | PN | T | HLV | E |
| RABV | KU888642 | F | PN | T | HLV | E |
| RABV | KU888643 | F | PN | T | HLV | E |
| RABV | KU888644 | F | PN | T | HLV | E |
| RABV | KU888645 | F | PN | T | HLV | E |
| RABV | KU899138 | F | PN | T | HLV | E |
| RABV | O92284 | F | PN | T | HLV | E |
| RABV | AB085828 | F | PN | T | HLV | E |
| RABV | EF206712 | F | PN | T | HLV | E |
| RABV | EF206711 | F | PN | T | HLV | E |
| RABV | EF206710 | F | PN | T | HLV | E |
| RABV | FJ959397 | F | PN | T | HLV | E |
| RABV | EU643590 | F | PN | T | HLV | E |
| RABV | FJ866836 | F | PN | T | HLV | E |
| RABV | FJ866835 | F | PN | T | HLV | E |
| RABV | EU877071 | F | PN | T | HLV | E |
| RABV | EU877070 | F | PN | T | HLV | E |
| RABV | EU877069 | F | PN | T | HLV | E |
| RABV | EU877068 | F | PN | T | HLV | E |
| RABV | EU877067 | F | PN | T | HLV | E |
| RABV | FJ712196 | F | PN | T | HLV | E |
| RABV | FJ712195 | F | PN | T | HLV | E |
| RABV | FJ712194 | F | PN | T | HLV | E |
| RABV | FJ712193 | F | PN | T | HLV | E |
| RABV | AB128149 | F | PN | T | HLV | E |
| RABV | EU311738 | F | PN | T | HLV | E |
| RABV | NC001542 | F | PN | T | HLV | E |
| RABV | EF206720 | F | PN | T | HLV | E |
| RABV | EF206719 | F | PN | T | HLV | E |
| RABV | EF206718 | F | PN | T | HLV | E |
| RABV | EF206717 | F | PN | T | HLV | E |
| RABV | EF206716 | F | PN | T | HLV | E |
| RABV | EF206715 | F | PN | T | HLV | E |
| RABV | EF206714 | F | PN | T | HLV | E |
| RABV | EF206713 | F | PN | T | HLV | E |
| RABV | EU293121 | F | PN | T | HLV | E |
| RABV | EU293113 | F | PN | T | HLV | E |
| RABV | EU293111 | F | PN | T | HLV | E |
| RABV | EU549783 | F | PN | T | HLV | E |
| RABV | EU293115 | F | PN | T | HLV | E |
| RABV | EF437215 | F | PN | T | HLV | E |
| RABV | EU293116 | F | PN | T | HLV | E |
| RABV | AB044824 | F | PN | T | HLV | E |
| RABV | AB009663 | F | PN | T | HLV | E |
| RABV | AY956319 | F | PN | T | HLV | E |
| RABV | EU182346 | F | PN | T | HLV | E |
| RABV | AY705373 | F | PN | T | HLV | E |
| RABV | EF206708 | F | PN | T | HLV | E |
| RABV | EF564174 | F | PN | T | HLV | E |
| RABV | EF206709 | F | PN | T | HLV | E |
| ABLV | GU936883 | F | PN | T | HLI | E |
| ABLV | KU739052 | F | PN | T | HLI | E |
| ABLV | NC003243 | F | PN | T | HLI | E |
| ABLV | AF081020 | F | PN | T | HLI | E |
| ARAV | EF614259 | F | PN | T | HMV | E |
| BBLV | KU761304 | F | PN | T | HLI | E |
| DUVV | EU623444 | F | PN | T | HLV | E |
| DUVV | GU936870 | F | PN | T | HLV | E |
| DUVV | KU739053 | F | PN | T | HLV | E |
| DUVV | KU761302 | F | PN | T | HLV | E |
| DUVV | EU293119 | F | PN | T | HLV | E |
| DUVV | EU293120 | F | PN | T | HLV | E |
| EBLV-1 | EU293112 | F | PN | T | HLV | E |
| EBLV-1 | EU352768 | F | PN | T | HLV | E |
| EBLV-1 | GU936874 | F | PN | T | HLV | E |
| EBLV-1 | KU739051 | F | PN | T | HLV | E |
| EBLV-1 | EU626552 | F | PN | T | HLV | E |
| EBLV-1 | EF157976 | F | PN | T | HLV | E |
| EBLV-1 | EU626551 | F | PN | T | HLV | E |
| EBLV-1 | NC009527 | F | PN | T | HLV | E |
| EBLV-2 | AAX62813 | F | PN | T | HLI | E |
| EBLV-2 | EU352769 | F | PN | T | HLI | E |
| EBLV-2 | GU936871 | F | PN | T | HLI | E |
| EBLV-2 | GU936882 | F | PN | T | HLI | E |
| EBLV-2 | KU761301 | F | PN | T | HLI | E |
| EBLV-2 | NC009528 | F | PN | T | HLI | E |
| EBLV-2 | EF157977 | F | PN | T | HLI | E |
| EBLV-2 | EU293114 | F | PN | T | HLI | E |
| IKOV | JX193798 | F | PD | T | ESI | E |
| IRKV | EF614260 | F | PN | T | HFV | E |
| IRKV | YP007641400 | F | PN | T | HFV | E |
| KHUV | EF614261 | F | PN | T | HLI | E |
| LBV | EF547428 | F | PN | T | HLI | E |
| LBV | EF547431 | F | PN | T | HLI | E |
| LBV | KU761303 | F | PN | T | HLI | E |
| LBV | EU293108 | F | PN | T | HLI | E |
| LBV | EU259198 | F | PN | T | HLI | E |
| LBV | EU293110 | F | PN | T | HLI | E |
| MOKV | GQ500108 | F | PN | T | HLI | E |
| MOKV | HM623780 | F | PN | T | HLI | E |
| MOKV | EU293118 | F | PN | T | HLI | E |
| MOKV | EU293117 | F | PN | T | HLI | E |
| MOKV | NC006429 | F | PN | T | HLI | E |
| SHIBV | NC025365 | F | PN | T | HLI | E |
| SHIBV | GU170201 | F | PN | T | HLI | E |
| WCBV | EF614258 | F | PN | T | NAI | E |

The eight positions important for CTB012 recognition of the rabies virus glycoprotein are highly conserved across 130 rabies and non-rabies lyssaviruses.
