# Supplementary material for: SYN023, a novel humanized monoclonal antibody cocktail, for post-exposure prophylaxis of rabies
Source: PLoS Negl Trop Dis. 2017 Dec 20;11(12):e0006133. doi: 10.1371/journal.pntd.0006133 (PMC5754141; doi:10.1371/journal.pntd.0006133)
Supplement: S2 Table — (DOCX) [file pntd.0006133.s006.docx]

**S2 Table. Neutralizing potency of CTB011, CTB012, and CTB011/CTB012 cocktails determined by RFFIT in different laboratories.**

| Sample No. | Sample Name | Potency (From AHA/USA)  IU/mL IU/mg | | Potency (From Synermore/China)  IU/mg IU/mg | |
| --- | --- | --- | --- | --- | --- |
| 011A | CTB011 | 182 | 1320 | 1633 | 1521 |
| 011B |  | 148 |  | 1410 |  |
| 012C | CTB012 | 93 | 744 | 698 | 699 |
| 012D |  | 93 |  | 701 |  |
| 111211E | CTB011/CTB012 cocktail (1:1) | 200 | 1744 | 1398 | 1484 |
| 111211F |  | 236 |  | 1570 |  |
| 111212G | CTB011/CTB012 cocktail (1:2) | 180 | 1380 | 1259 | 1264 |
| 111212H |  | 165 |  | 1268 |  |
| 111213I | CTB011/CTB012 cocktail (1:3) | 157 | 1260 | 1470 | 1368 |
| 111213J |  | 158 |  | 1266 |  |
| 111221K | CTB011/CTB012 cocktail (2:1) | 226 | 1764 | 1469 | 1686 |
| 111221L |  | 215 |  | 1902 |  |
| 111223M | CTB011/CTB012 cocktail (3:1) | 201 | 1664 | 1730 | 1556 |
| 111223N |  | 215 |  | 1383 |  |

The ratios represent mass ratios of CTB011:CTB012 adding up to the same total IgG concentration for all mAb cocktails.
